# Improved Patient Dosimetry at Radioiodine Therapy by Combining the ICRP Compartment Model and the EANM Pre-Therapeutic Standard Procedure for Benign Thyroid Diseases

**DOI:** 10.3389/fendo.2021.634955

**Published:** 2021-03-12

**Authors:** Martin Andersson, Sören Mattsson

**Affiliations:** ^1^ Department of Radiation Physics, Institute of Clinical Sciences, Sahlgrenska Cancer Center, Sahlgrenska Academy, University of Gothenburg, Gothenburg, Sweden; ^2^ Medical Radiation Physics, Department of Translational Medicine Lund University, Malmö, Sweden

**Keywords:** dosimetry—radiation, radioiodine (131I) treatment, hypothyroidism, hyperthyroidism (Graves’ disease), hyperthyroidism—diagnosis, radioiodine

## Abstract

Radioactive iodine is commonly used for the treatment of different thyroid conditions since the 1940s. The EANM has developed a standard pre-therapeutic procedure to estimate patient specific thyroid uptake at treatment of benign thyroid diseases. The procedure which models the time dependent fractional thyroid uptake is based on a two-compartment fitting system, one representing the thyroid and the other the blood. The absorbed dose is however only estimated for the thyroid and not for any other organ in the body. A more detailed biokinetic model for iodine is given by the ICRP and includes an iodide transport in the whole body. The ICRP model has 30 different compartments and 48 transfer coefficients to model the biokinetics of iodide and to model different transfer for inorganic iodide and organic iodine. The ICRP model is a recirculation iodine model, and the optimization is performed on the whole model and not exclusively on the thyroid as in the EANM procedure. Combining the EANM method and the ICRP model gives both patient specific estimations of thyroid uptake and retention and include most organs in the body. The new software gives both an improved patient specific dosimetry for the thyroid and an estimation of the absorbed dose to non-target organs and tissues like kidneys, urinary bladder, stomach wall, and uterus. Using the method described in this paper, the repercussions on the daily routines will be minimal.

## Introduction

Radioactive iodine is commonly used for the treatment of benign thyroid diseases and of various forms of thyroid cancer since the 1940s (1–4). This is due to the fact that iodine is an essential component in the production of the thyroid hormones thyroxine (T4) and triiodothyronine (T3), which regulate metabolic processes and are critical to growth and developments. The dosimetry of radioactive iodide, the ionic form of iodine, is of interest for occupationally exposed workers, for environmental exposure, and for nuclear medicine diagnostics as well as therapy. Therefore, various biokinetic models have been developed during the years for different purposes and iodine isotopes (5–12). For instance, the International Commission on Radiological Protection (ICRP) previously had two different biokinetic models for iodide, one for nuclear medicine (13) and another for occupational (14) exposure to estimate the distribution in different organs and tissues. However, in the transition to systemic biokinetic compartment modeling, the transfer of iodide in the whole human body is now treated in the same way for both nuclear medicine (15) and occupational exposure (16) and based on the biokinetic models of Leggett (11, 12) to calculate the absorbed dose and the effective dose.

For nuclear medicine four isotopes of iodine are of main interest. The most commonly used in both diagnostic and therapeutic nuclear medicine is I-131, while I-123, I-124, I-125 are radioisotopes only for diagnostic purposes (10). In nuclear medicine radioactive iodine in the form of I-131 iodide is used to treat patients either with hyperthyroidism by destroying a sufficient amount of thyroid tissue to render the patient either euthyroid or hypothyroid or in the case of thyroid cancer treatment eliminating residual thyroid cancer tissue as well as local and distant microscopic disease following thyroidectomy.

Nevertheless, it is stated that treatment of patients using radiopharmaceuticals should be based on individually planned pre-therapeutic dosimetry (17–19). This dosimetry should determine the most likely amount of activity which leads to a therapeutic success in the target volume and giving non-target volumes and tissues a low as reasonably achievable exposure (20). For this pre-therapeutic dosimetry, patients are administered with a low test activity of I-131 and followed by one to three fractional iodide uptake measurements over the thyroid using a dedicated probe or by planar scintigraphy (21).

To help medical physicist, scientists, and clinicians on how to perform patient-specific absorbed dose assessments, the EANM Dosimetry Committee has published a standard operational procedure for pre-therapeutic dosimetry and absorbed dose calculations (22). However, the EANM model only focuses on estimating patient specific absorbed dose to the thyroid, while the ICRP iodide model is a generic model, which estimates the absorbed dose to all organs.

### EANM Pre-Therapeutic Standard Procedure

In the EANM standard procedure, the fractional I-131 uptake in the thyroid at time t, is called RIU(t). The RIU(t) includes physical decay, meaning no decay correction is applied if I-131 is used for pre-therapy dosimetry making the quantity RIU(t) to directly reflect the kinetics of I-131 in the thyroid tissue (22). By determining the RIU(t) in the thyroid over the whole iodide treatment period, an administered activity, which reaches a therapeutic goal could be determined by:

$$A_{a}=\frac{1}{\bar{E}}*\frac{M*D}{\int_{0}^{\infty }RIU\left(t\right)dt}$$

where *A_a_* is the activity to be administered for therapy, *Ē* is the mean energy deposited per decay of I-131 in the thyroid, *M* is the mass of the patient´s thyroid, *D* is the prescribed mean absorbed dose to the thyroid to reach a therapeutic successful outcome.

**Figure 1:**
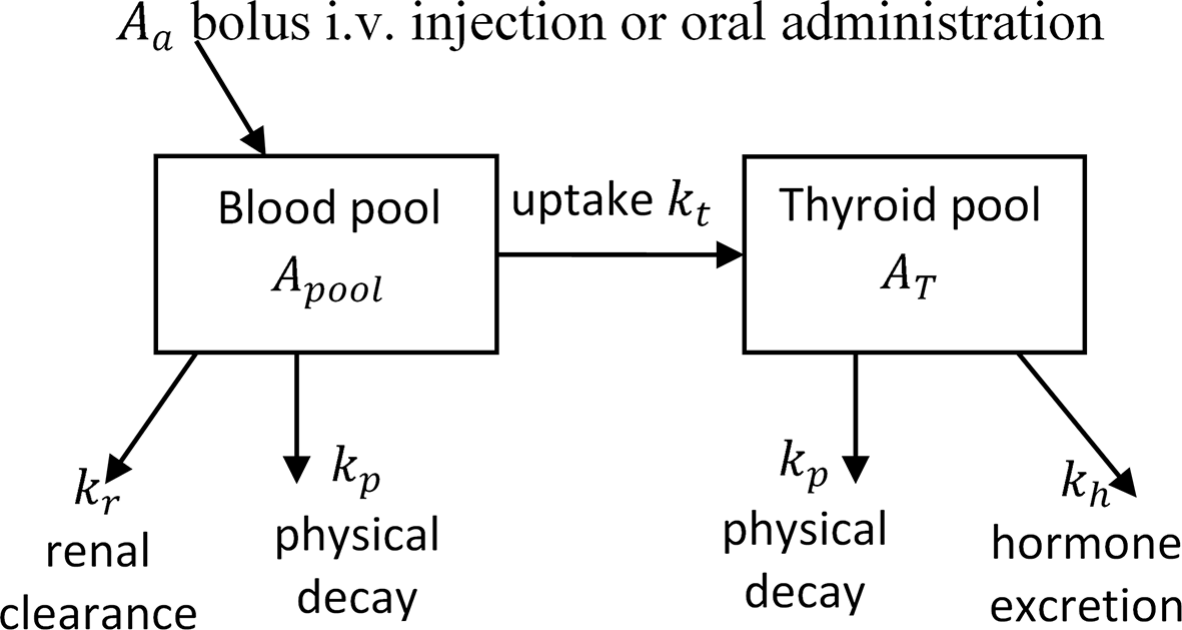
The EANM compartment model to estimate the activity in the thyroid. The EANM model is a two-compartment model, which uses the blood pool as supporting compartment to determine the thyroid activity.

The therapeutic activity can be estimated by different methods based on one to three activity uptake measurements over the thyroid. For the one and two measurements alternative, many simplifications of the thyroid uptake are included to be able to estimate an administered activity *A_a_* resulting in the prescribed absorbed dose. As the accuracy increases with increasing time of the measurement after the administration of I-131, the uncertainties are lower with a measurement after 48 h than with a measurement after 24 h. Therefore, a two-compartment model including at least three uptake assessments at about 4 to 6 h, 1 to 2 days, and 5 to 8 days after activity administration is recommended by the EANM (22). This recommended two-compartment model is shown in **Figure 1**, and by modifying the transfer coefficients for the thyroid uptake, *k_t_*, the renal clearance, *k_r_*, and the hormone excretion, *k_h_*, to the RIU(t) patient time specific uptake assessments, the total number of I-131 disintegration in the thyroid could be determined. This determination, based on three or more unique activity assessments of three unknown parameters (*k_t_*, *k_r_*, *k_h_*) without any recommended parameter intervals of constrains, is a mathematical problem which will have a unique solution. The EANM model is based on using the time dependent activity in the compartment blood pool, *A_pool_* to support the compartment modeling of I-131 activity in the thyroid.

### ICRP Compartment Model

**Figure 2:**
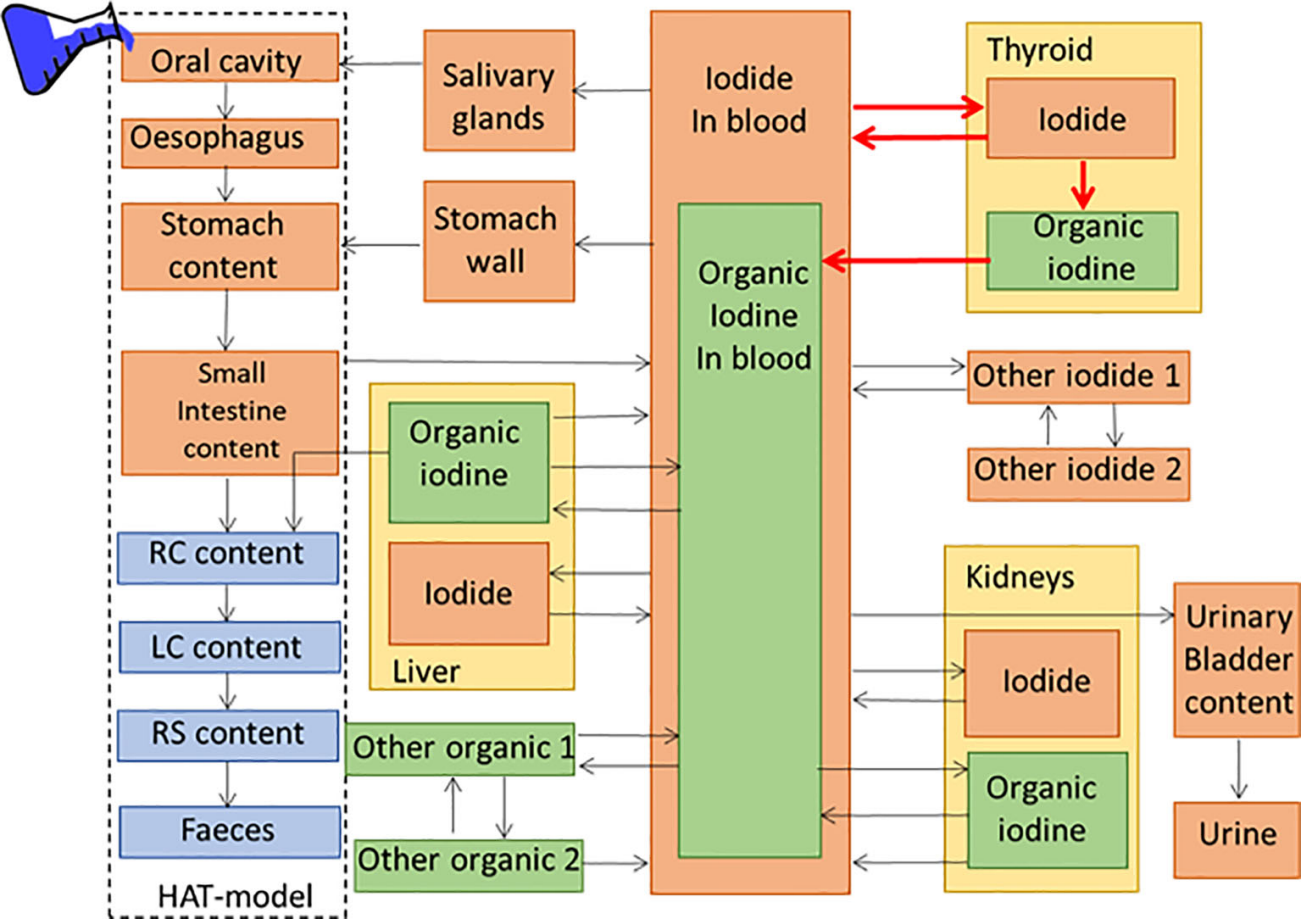
The ICRP compartment model for oral administration of iodide includes 30 different compartments and 48 transfer coefficients to model the bio-distribution of iodide in the human body. The transfer coefficients marked in red can be fitted to the patient specific uptake.

In the ICRP biokinetic compartment model for iodine, the daily intake of stable iodine set to 160 μg. The biokinetic compartment model for iodide includes 30 different compartments and 48 transfer coefficients to model the biokinetics of iodide in the human body. Of these are two compartments used to describe the iodide uptake and retention in the thyroid, one for inorganic iodide and one for organic iodide, with four transfer coefficients. The biokinetic model for orally administered activity is shown in **Figure 2**. Applying the transfer coefficients based on a daily intake of 160 μg iodine results in a 26% uptake of I-131 in the thyroid 24 h after administration. However, patient treated with iodide can both deviate from the normal hormone levels of T3, and T4 and the daily iodide intake. Therefore, the ICRP also has included two additional biokinetic models with a higher and a lower transfer coefficient from iodide in blood to iodide in thyroid which corresponds to a 24-h uptake of 16 and 36%, respectively. As for the blocked thyroid cases, this corresponds to an assumption of no transfer of iodide to organic iodine, by setting the transfer coefficient from thyroid iodide to organic iodide in the thyroid to 0 (15). For orally administered activities, the activity is placed in the compartment representing the oral cavity, and for intravenously administered activity, the initial activity is placed in the compartment representing the iodide in blood.

The aim of this project is to combine the EANM method and the ICRP model. This could give both a patient specific dosimetry for the thyroid, based on the whole body transfer of both inorganic iodide and organic iodine and a possibility to estimate the absorbed dose to non-target organs and tissues—in both cases using the latest iodide biokinetic information. The repercussions on the existing daily routines will also be evaluated.

## Method

The combination of the EANM method and the ICRP model is performed by adjusting the four transfer coefficients in the ICRP model, which regulates the iodide and iodine uptake in the thyroid, to the result of patient specific measurements and are marked in red in **Figure 2**. The fitting will be performed on the fractional *RIU*(*t)* data points over the whole thyroid, including the activity of both compartments—one for iodide and one for organic iodine. The fitting can be performed on both intravenously or orally administered activity with the difference that the initial activity is either placed in the oral cavity or in blood. For adjusting the ICRP model for the blocked thyroid case, the transfer coefficient from thyroid iodide to organic iodine in the thyroid is set to 0, making only modification of the two inorganic transfer coefficients. The curve fitting can be performed on either one or all four transfer compartments, with arbitrary restrains on the transfer coefficients. The fitting is based on a cost function with minimizing the total square distances between the fitted activity parameters and the measured data points. The fitting can be performed on one or several measurements.

**Figure 3:**
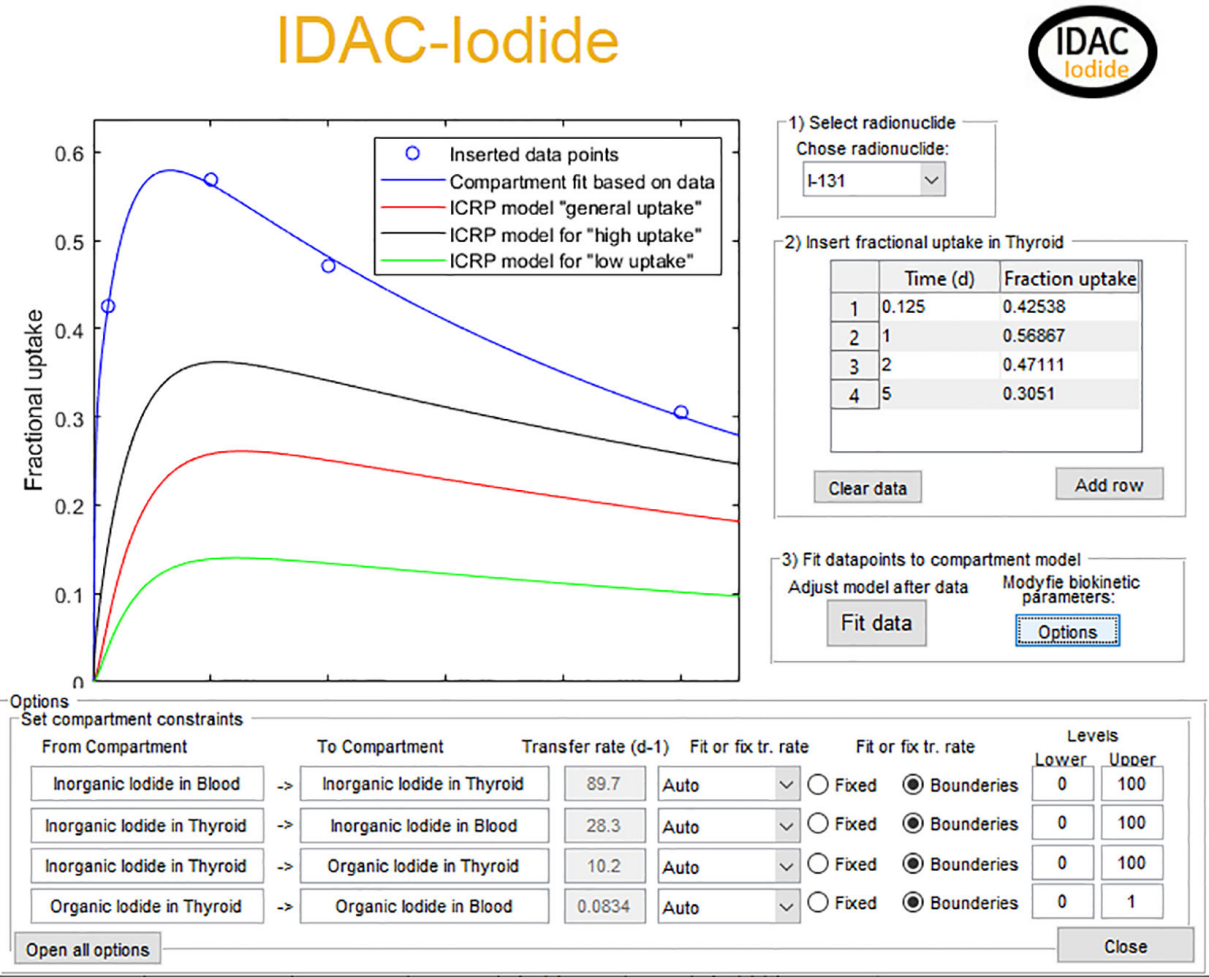
The graphical input interface of the software. The blue circles are measured fractional uptake activity data of I-131 for a hyperthyroid patient. The red line in the ICRP biokinetic model with general uptake and the black and green line is the corresponding ICRP model for high and low uptake, respectively. The blue line is the combined EANM/ICRP method with the transfer of inorganic uptake from blood to thyroid modified after the patient data.

The I-131 activity for the normal and high thyroid uptake is shown in **Figure 3**, together with the results of activity measurements of an adult Graves’ patient. As shown in **Figure 3**, the high thyroid uptake is not always representative for the specific patients. The blue line is the thyroid uptake of modified ICRP model using the EANM pre-therapeutic model with modification of the transfer coefficients. The ICRP iodide biokinetic model is also an age specific model, making it possible to use any of the age specific (adult, 15-yrs, 10-yrs, 5-yrs, 1-yr, or 100 days) biokinetic models the fitting of patient data.

### Twenty-Two Isotopes of Radioiodine

The by far most commonly used iodine isotope is I-131, but the ICRP biokinetic model is valid for all iodine isotopes with a half-life longer than 15 min, progenies included (16). Therefore, there is also a possibility to change the biokinetic fitting and dosimetric calculations to any of the 22 radioisotopes of iodine. Biokinetic and dosimetry calculation of possible radioactive daughter nuclides are included. The model also enables biokinetic and dosimetric estimations for population groups or individuals outside nuclear medicine.

### Absorbed Dose and Effective Dose Calculations

The absorbed dose calculations follow the ICRP computational framework of occupational intake given in ICRP Publication 130 (23) and the specific absorbed fractions of ICRP Publication 133 (24). To estimate the radiation risk, the mean absorbed dose to a target region was calculated by (25)

$$D\left(r_{T},T_{D}\right)=\underset{r_{s}}{\sum }\overset{\infty }{\underset{0}{\int }}A\left(r_{s},t\right)*S\left(r_{T}\leftarrow r_{s,t}\right)dt\left[\text{Gy}\right]$$

where *A*(*r_s_*, *t*) is the time dependent activity at time *t*, in source region *r_s_* from administration to ∞, *S*(*r_T_* ← *r_s_*) is the mean absorbed dose in target *r_T_* per nuclear transformations in source region *r_s_*. The *S*(*r_T_* ← *r_s_*) is generated with radionuclide decay scheme and Monte Carlo simulated specific absorbed fractions by simulate every source-target combination

$$S\left(r_{T}\leftarrow r_{s}\right)=\underset{i}{\sum }\Delta_{i}\Phi \left(r_{T}\leftarrow r_{s},E_{i}\right)\left[\text{Gy}/\text{Bq}\right]$$

where *Φ*(*r_T_* ← *r_s_*, *E_i_*) is the absorbed fraction from the source region to the target region *r_T_* divided by the mass in kilograms of the target region *r_T_* of the *i*th components in the decay scheme and *Δ_i_* = *E_i_Y_i_* is the energy yield there *Y_i_* is the yield and *E_i_* is the mean energy of the *i*th nuclear transition of the radionuclide in joule.

The effective dose (*E*) (26), which is the sum of sex average radiation weighted equivalent dose from radiosensitive organs is calculated by

$$E=\sum_{T}w_{T}H_{T}=\sum_{R}\frac{w_{R}D_{T,R_{Ref,male}}+w_{R}D_{T,R_{Ref,female}}}{2}[Sv]$$

where *w_R_* is the radiation weighting factors of radiation *R*, *w_T_* is the tissue weighting factor representing the relative organs and tissues detrimental effects. *D*(*r_T_*, *T_D_*)*_Male_* and *D*(*r_T_*, *T_D_*)*_Female_* is the mean absorbed dose of target region *T* of the reference male and female person, respectively.

The effective dose is calculated according to ICRP Publication 103 and with an additional calculation with a tissue weighting factor for thyroid set to zero, *w_T,Thyroid_* = 0, as the radioiodine treatments will create cell killing in the thyroid. This additional calculation gives a risk indicator for the non-target organs and tissues.

### Modification of Thyroid Mass for Self-Irradiation

The dose calculations are performed for the ICRP adult voxel phantoms which have a thyroid mass of 23.4 g for the male and 19.5 g for the female (24). If the patient’s thyroid deviates from this mass, a modification of the self-irradiation for thyroid can be performed. This mass correction will for photons be performed by adding the factor (*M_ICRP_*/*M_pat_*)^2/3^, where *M_ICRP_* is the mass of the reference phantoms and *M_pat_* is the new organ mass of the patient (27). For particles, this factor is *M_ICRP_*/*M_pat_*.

All biokinetic modelling and dosimetric assumptions and calculations can also be saved to a PDF dose report, which includes all data used in the calculations.

### Iodide Uptake in the Thyroid of 15 Hyperthyroid Patients

During 1984–1989 a database for 516 consecutive patients treated for hyperthyroidism at Malmö University Hospital was created. As a base for the treatment four pre-therapeutic uptake measurements were made for each patient 3, 24, 48 h, and a fourth measurement between 3 and 9 days (17, 21, 28). The iodide uptake measurements in the thyroid were made using a 5 cm (diam.) × 5 cm NaI(Tl)-detector equipped with a standard thyroid uptake collimator (29) and a thyroid phantom (30). Out of these 515 patients, a group of 15 patients with hyperthyroidism were randomly selected to illustrate and test the proposed method.

### Framework for Dose Assessment

The generated software provides an easy way or estimate patient specific thyroid uptake, based on one or preferably several fractional I-131 uptake values. The software adjusts the biokinetic parameters to fit the time activity curves on the measured data points. To perform patient specific dose estimations, the mass of the thyroid is included in the calculation. The presented framework is thus based on only two different patient parameters, thyroid uptake and mass, which makes it a fast and easy method for the clinical routine to perform dose assessment for the thyroid and non-target organs and tissues.

## Results and Discussion

**Figure 4:**
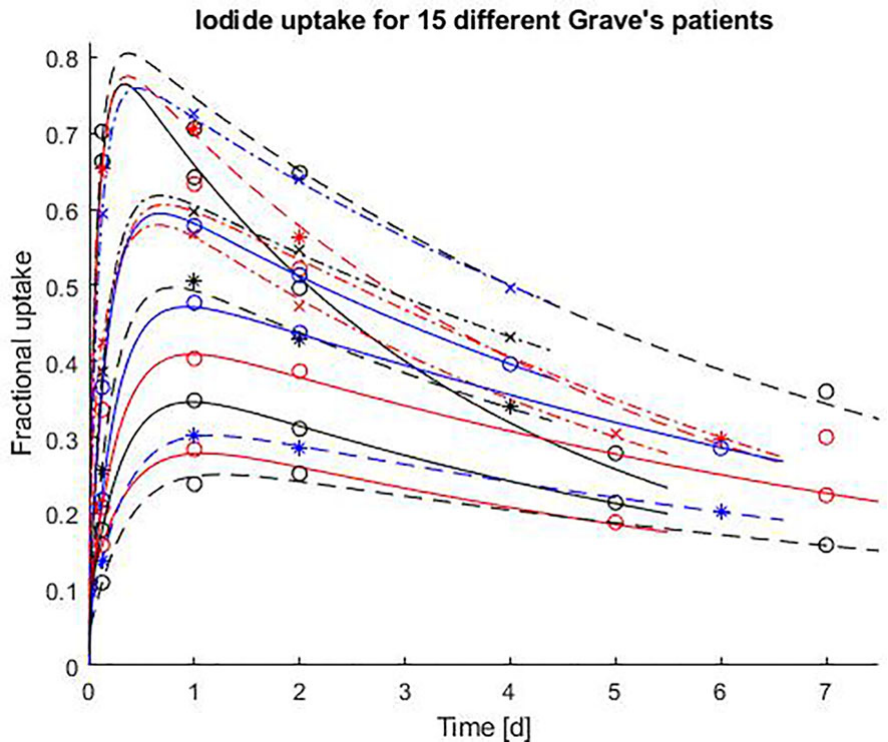
Fractional thyroid uptake of I-131 iodide as a function of time after a pre-therapeutic administration in 15 hyperthyroid patients. The results of the uptake measurements are marked as “o”, “x”, “*”. The solid and dashed lines are the patient specific time activity curves of I-131, generated by the software described in this paper, and based on the four uptake measurements.

The mathematical code was tested on measured activity data for 15 I-131 hyperthyroid patients. The fractional thyroid uptake of I-131 iodide as a function of time after a pre-therapeutic administration in 15 hyperthyroid patients are shown in **Figure 4**. The results show that for each 15 randomly selected patients there is a good agreement between the generated time dependent uptake/retention curve and all four measurement points.

For the 15 I-131 hyperthyroid patients, the mean time-integrated activity coefficient (TIAC) for thyroid was estimated to 113 MBq h/MBq (range of 67–170) giving a female thyroid dose coefficient of 653 mGy/MBq (range 390–981). Giving a factor of 2.5 difference within the patient cohort. This strongly shows the importance of patient specific dose planning. To estimate dose coefficients for organs and tissues outside the thyroid, the software calculates sex specific organ doses, which are of concern for stochastic effects. **Table 1** shows the female organ dose coefficients of thyroid, stomach wall, kidneys, urinary bladder wall, and uterus. For some organs the dose contribution from the treatment is not negligible. A typical administered activity of 300 MBq (28) gives an absorbed dose to the kidneys of 231 mGy (range 48–597), to the stomach wall 114 mGy (range 96–168), urinary bladder wall 45 mGy (range 36–69), and uterus 33 mGy (range 18–72). The dose data for the uterus is needed in the case of early unexpected early progeny.

**Table 1:** Thyroid TIAC, effective dose with and without thyroid contribution and absorbed doses for selected female organs.

Id	Thyroid TIAC[MBq-h/MBq]	Effective dose [mSv/MBq]		Female organ absorbed doses [mGy/MBq]				
		*ED*	$ED_{wt_{(thyroid)=0}}$	*Thyroid*	*Stomach wall*	*Kidneys*	*Urinary bladder wall*	*Uterus*
**1**	107	23	0.53	620	0.43	1.05	0.17	0.14
**2**	148	32	0.54	854	0.32	0.57	0.11	0.09
**3**	107	23	0.46	622	0.38	0.67	0.14	0.11
**4**	170	37	0.64	981	0.32	0.81	0.12	0.11
**5**	128	28	0.53	742	0.36	0.76	0.14	0.11
**6**	98	21	0.70	564	0.56	1.99	0.23	0.24
**7**	67	15	0.30	390	0.39	0.38	0.15	0.09
**8**	75	16	0.35	431	0.40	0.54	0.15	0.10
**9**	89	19	0.32	517	0.34	0.19	0.12	0.06
**10**	125	27	0.68	724	0.47	1.57	0.19	0.19
**11**	114	25	0.43	658	0.35	0.47	0.13	0.08
**12**	116	25	0.53	669	0.40	0.94	0.16	0.13
**13**	77	17	0.03	446	0.35	0.16	0.13	0.06
**14**	106	23	0.39	616	0.34	0.35	0.12	0.07
**15**	166	36	0.68	961	0.35	1.05	0.13	0.13
**Mean** **(min–max)**	113 (67–170)	24 (15–37)	0.47 (0.03–0.70)	653 (390–981)	0.38 (0.32–0.56)	0.77 (0.16–1.99)	0.15 (0.12–0.23)	0.11 (0.06–0.24)

**Figure 5:**
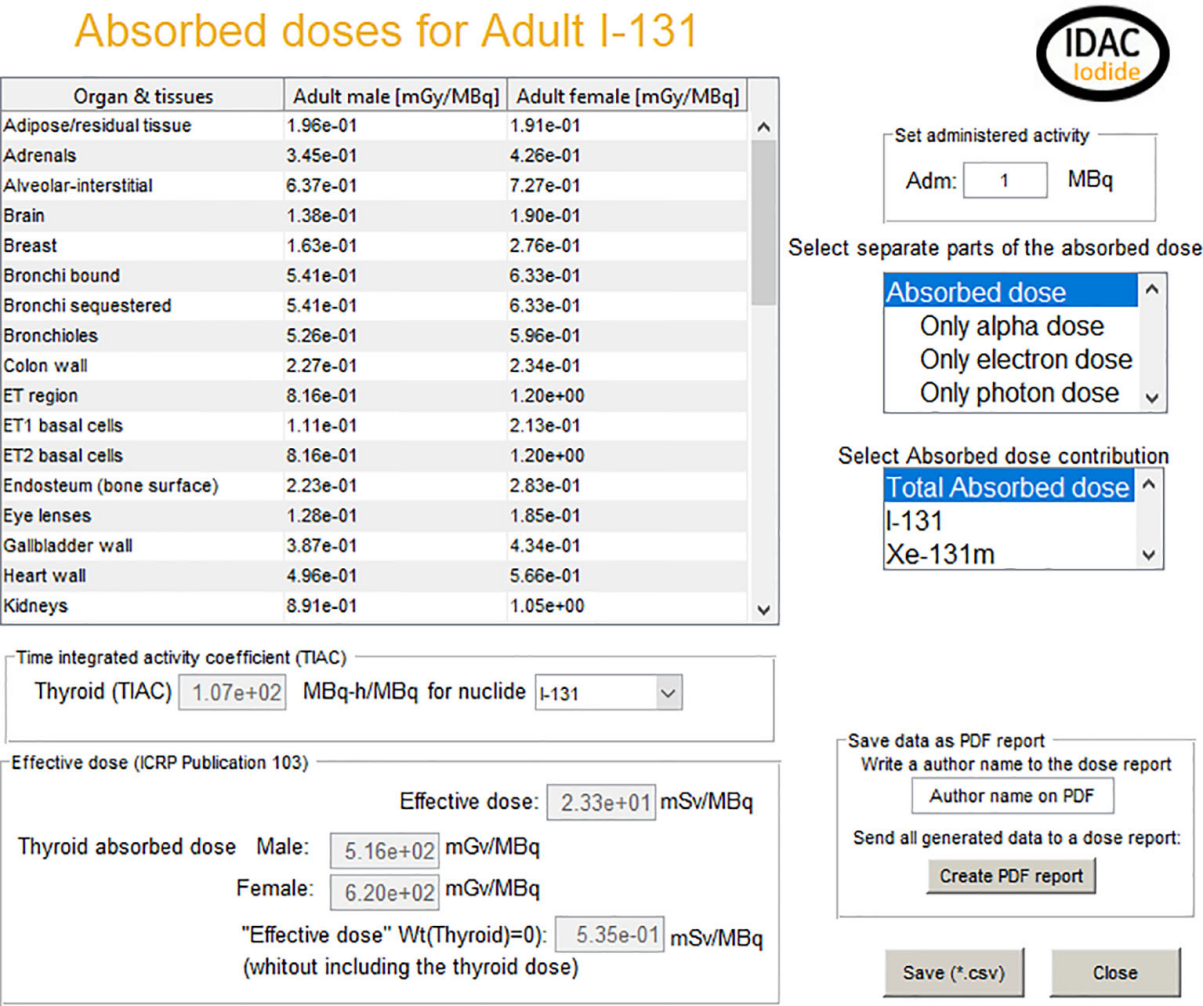
The graphical interface for the output of the calculations. It includes absorbed dose to organs and tissues, the effective dose, and the thyroid time-integrated activity of the fit described in **Figure 3**.

The transfer coefficient for patient Id 1 (**Table 1**), from blood to inorganic thyroid, inorganic thyroid to blood, inorganic thyroid to organic thyroid, and organic thyroid to blood was changed from 7.3, 36, 95, and 0.0077 day^−1^ as in the ICRP normal uptake model to 55, 12, 8.9, and 0.083 day^−1^, respectively. Giving in the ICRP normal uptake model a 1.4 times higher thyroid uptake, and the iodide thyroid uptake is shown in **Figure 3**. The increased thyroid uptake from blood will decrease the excretion to feces and urine and 1.8 times more I-131 will decay within the body. This increased thyroid uptake will also result in a 1.4 times higher thyroid absorbed dose (from 365 to 516 mGy/MBq). For all other radiosensitive organs, the absorbed dose will in average increase with a factor of 1.4, when adjusting the biokinetic model to the measured patient data. The graphical output of the code, for patient Id 1, is shown in **Figure 5**. The absorbed dose is shown for both the I-131 and its decay product Xe-131m. However, the absorbed dose contribution from Xe-131m is neglectable and not often included in iodine I-131 calculations. As the code can include 22 different radioisotopes of iodide and where in some cases the decay products with their specific biokinetics could have a significant contribution to the total absorbed dose, decay products are included in the program.

The results also give the thyroid specific time integrated effective dose coefficient, allowing the use of only the biokinetic part of the code to be exported to external absorbed dose calculation software. In the case of patient Id 1, the effective dose 23.3 mSv per MBq where 97.5% of the effective dose contribution comes from the absorbed dose to other organs than the thyroid. For the surrounding non-target organs are therefore, an additional calculation performed excluding the thyroid contribution performed. This calculation shows a 0.53 “mSv/MBq” which could be used as a compliment to the specific organ absorbed dose values given in the table in **Figure 5**.

The proposed method gives both the individually planned pre-therapeutic dosimetry for treatments of thyroid patients to reach the therapeutic goal and also to control the absorbed dose to the non-target organs and tissues. The patient specific biokinetic modifications can be performed based on only one uptake measurement. However, the three uptake assessments at about 4 to 6 h, 1 to 2 days, and 5 to 8 days after activity administration recommended by the EANM provides a more accurate dosimetry. In the case that only one activity measurement of iodide in the thyroid is possible 4 days after administration is preferable (21).

## Conclusion

The EANM pre-therapeutic standard procedure for benign thyroid diseases and the ICRP biokinetic and dosimetric model for iodine have been combined in a specific software. It was tested on measured thyroid uptake and retention data for I-131 iodide in 15 randomly selected hyperthyroid patients. The fractional thyroid uptake of I-131 iodide as a function of time after a pre-therapeutic administration shows that for each 15 patients there is a good agreement between the generated time dependent fractional thyroid uptake/retention curve and all four measurement points. The new software gives both an improved patient specific dosimetry for the thyroid and an estimation of the absorbed dose to non-target organs and tissues like kidneys, urinary bladder, stomach wall, and uterus. The software gives possibilities to perform advanced dose estimations in a standardized and simple way, with the minimum of input parameters. Using the software developed and described in this paper, the repercussions on the daily routines will be minimal.

## Dedication

Dedicated to the memory of our late colleague and friend, Professor Lennart Johansson (1951–2020) with gratitude for many years of very fruitful cooperation.

## Data Availability Statement

The raw data supporting the conclusions of this article will be made available by the authors, without undue reservation.

## Author Contributions

MA wrote the code and developed the software. Both SM and MA participated in the study planning, data analysis, and drafting of the manuscript. All authors contributed to the article and approved the submitted version.

## Conflict of Interest

MA has created a webpage and a distribution channel IDAC-Dose AB to be able to distribute the software generated in this article.

The remaining author declares that the research was conducted in the absence of any commercial or financial relationships that could be construed as a potential conflict of interest.
